# Anxiety sensitivity and intolerance of uncertainty track distinct neurobehavioral dimensions of avoidance in anxiety-related disorders

**DOI:** 10.1038/s41380-026-03640-1

**Published:** 2026-05-27

**Authors:** Hannah Berg, Abigail Emich, Samuel E. Cooper, Christopher Hunt, Ryan D. Webler, Adrienne B. Manbeck, Abbey Hammell, Philip C. Burton, Scott R. Sponheim, Matt G. Kushner, Kendrick N. Kay, Shmuel Lissek

**Affiliations:** 1 University of Minnesota – Twin Cities, Minneapolis, MN USA; 2 Laureate Institute for Brain Research, Tulsa, OK USA; 3 University of Texas at Austin Dell Medical School, Austin, TX USA; 4 University of California – San Diego School of Medicine, San Diego, CA USA; 5 Brigham and Women’s Hospital, Harvard Medical School, Boston, MA USA; 6 Minneapolis Veterans Affairs Health Care System, Minneapolis, MN USA; 7 University of Minnesota – Twin Cities Medical School, Minneapolis, MN USA

**Keywords:** Psychiatric disorders, Neuroscience, Psychology

## Abstract

Avoidance behavior is a prominent and impairing feature of anxiety disorders, trauma- and stressor-related disorders, and obsessive-compulsive disorder. A transdiagnostic approach to identifying the neural basis of avoidance is a promising avenue to shed light on the heterogeneity among individuals affected by these conditions, collectively termed anxiety-related disorders (ARDs). In this cross-sectional study, 58 adults with ARDs and 77 healthy comparisons (HC) completed self-report measures of anxiety sensitivity (i.e., “fear of fear”) and intolerance of uncertainty (i.e., “fear of the unknown”), two transdiagnostic correlates of ARDs. Participants then completed an avoidance task during functional magnetic resonance imaging (fMRI), involving a threat cue paired with shock, safety cues, and safe generalization stimuli with varying resemblance to the threat cue. We examined neural activity preceding avoidance decisions in two phases: a) threat reactivity and b) mental simulation. Threat reactivity in anterior insula and dorsomedial prefrontal cortex tracked threat-relevance of stimuli, as expected. ARD status and anxiety sensitivity both strengthened the relationship of threat reactivity in right and left anterior insula with maladaptive avoidance. We then applied multi-voxel pattern analysis (MVPA) to decode avoidance behavior from neural activity during mental simulation. For those with higher intolerance of uncertainty, avoidance behavior was less concordant with neural activity in motor cortex and intraparietal sulcus. Our results suggest that anxiety sensitivity and intolerance of uncertainty differentially alter the neural mechanisms of avoidance behavior in anxiety-related psychopathology, potentially warranting distinct intervention strategies.

## Introduction

**Fig. 1:**
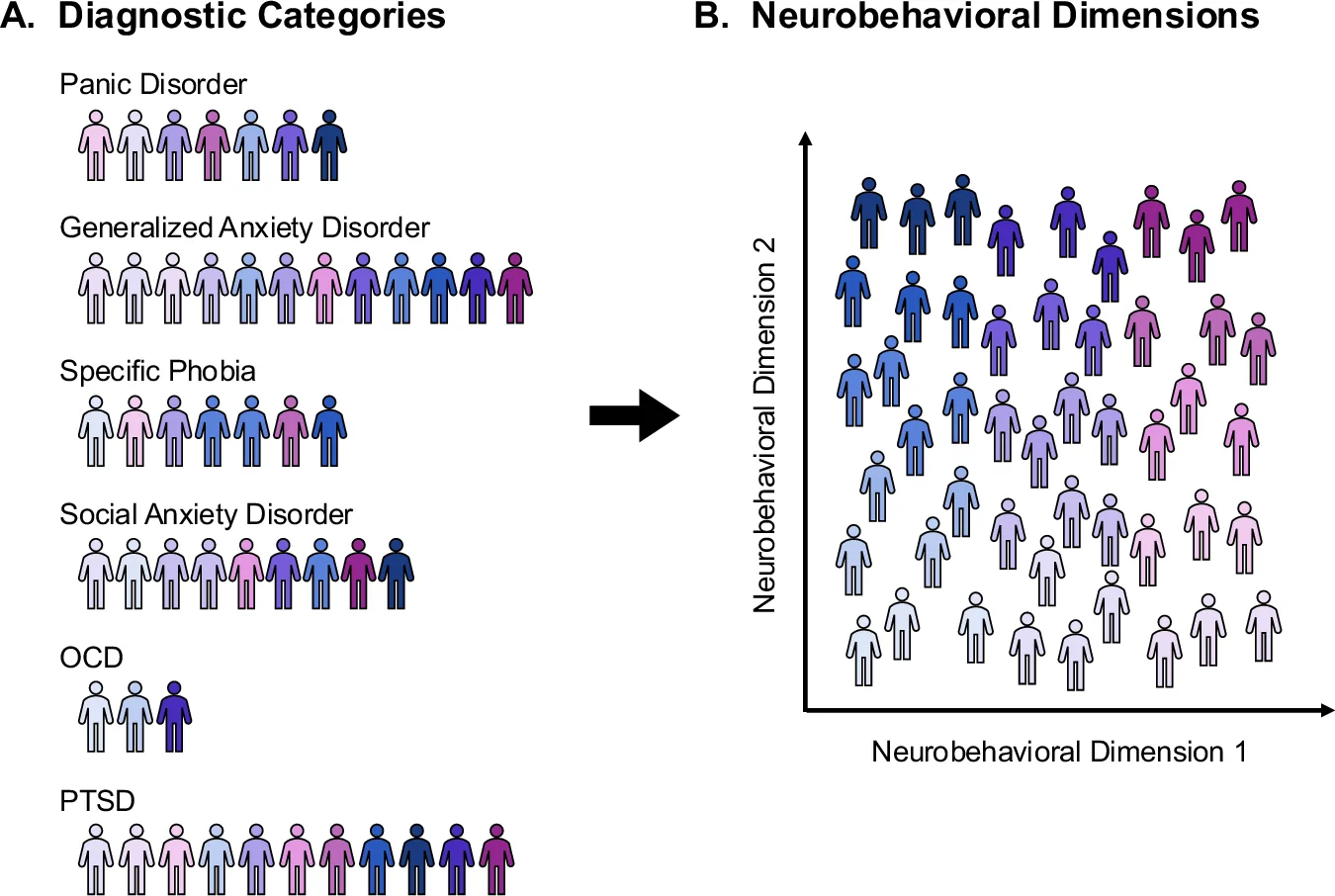
Conceptual rationale for examining avoidance in a transdiagnostic sample. **A** Existing diagnostic categories for anxiety-related disorders may not adequately capture the heterogeneity of these conditions. Some examples of anxiety-related disorders are shown here for illustrative purposes. **B** Avoidance behavior is also heterogeneous. For individuals with anxiety-related disorders, one or more specific contexts or brain states may tend to elicit avoidance. This simplified diagram illustrates neurobehavioral dimensions on which each individual can be placed, representing the extent to which particular contexts and brain states lead to avoidance behavior. Identifying clinically-relevant neurobehavioral dimensions is a promising avenue in the effort to improve conceptualization and treatment of anxiety-related psychopathology.

Avoidance is a core feature of psychiatric conditions marked by anxiety, including anxiety disorders, trauma- and stressor-related disorders, and obsessive-compulsive disorder [1]. Because anxiety and avoidance cut across these conditions, they are often grouped together as *anxiety-related disorders (ARDs)* [2]. Across ARDs, avoidance exacerbates symptoms, contributes to functional impairment, and is a crucial target for treatment [3–6]. Although the clinical expression of avoidance can differ across diagnoses, converging evidence suggests that these conditions share overlapping avoidance-related processes [7]. This transdiagnostic conceptualization is supported by preclinical work implicating common neural mechanisms of avoidance [8], and by the efficacy of exposure-based interventions that target avoidance across ARDs [9]. Together with evidence for a dimensional, rather than categorical, structure of anxiety symptoms [10, 11], these findings suggest that current diagnostic categories may not adequately capture the heterogeneity of avoidance across ARDs [12, 13]. Characterizing individual differences in the neurobehavioral mechanisms underlying avoidance may therefore improve the conceptualization and treatment of ARDs (Fig. 1).

Avoidance can be maladaptive when it is *costly* and/or *unnecessary*. Costly avoidance involves relinquishing rewards or valued outcomes (i.e., approach-avoidance conflict), whereas unnecessary avoidance occurs in response to objectively safe cues or situations. Laboratory studies show elevated costly avoidance across ARDs, including social anxiety disorder, panic disorder, and agoraphobia [14, 15], as well as posttraumatic stress disorder [16]. Experimental work also indicates heightened unnecessary avoidance to safe stimuli in panic disorder, posttraumatic stress disorder, and obsessive-compulsive disorder [17]. One mechanism by which such unnecessary avoidance may proliferate is fear generalization from a learned threat cue to a range of safe stimuli or situations that resemble the threat cue [18, 19]. Consistent with this account, meta-analytic evidence indicates elevated fear generalization across ARDs, particularly in generalized anxiety disorder, social anxiety disorder, and posttraumatic stress disorder [20]. Together with laboratory studies linking generalized fear to avoidance behavior [e.g., 21–28], these findings implicate fear generalization as a plausible driver of maladaptive avoidance in ARDs. Here, we aimed to identify the neural correlates of fear generalization and the accompanying costly, unnecessary avoidance in well-characterized ARD sample.

One candidate mechanism of avoidance behavior is initial *threat appraisal*, involving orienting to and reactivity toward threat. Prior neuroimaging meta-analyses indicate that learned threat cues elicit conditioned (i.e., Pavlovian) responses in anterior insula, dorsal anterior cingulate cortex (dACC) and dorsomedial prefrontal cortex (dmPFC) [29], and that this activity generalizes across safe stimuli resembling the threat cue [30]. These regions are functional hubs of the task-positive salience network [31, 32], supporting the notion that they subserve initial orienting toward threats. Another meta-analysis identified heightened activity in these regions among patients with ARD relative to healthy comparison participants across a variety of affective task paradigms [33], implicating hyperactive salience network activity as a transdiagnostic feature of ARDs. By contrast, the ventromedial prefrontal cortex (vmPFC) has been implicated in fear *inhibition*, with activity that decreases with threat-relevance of stimuli [30] and increases with safety learning [34].

Following initial threat appraisal, individuals can further optimize behavior by engaging in *mental simulation* and evaluation of potential outcomes [35–37]. Human neuroimaging research has identified substantial overlap between the neural substrates of simulating possible future events and remembering past events [38–40], centering on the hippocampus, vmPFC and posterior cingulate cortex (PCC) [38, 39, 41–45]. These regions are also functional hubs of the default network (DN), whose activity is thought to subserve the mental simulation of past and imagined events that occurs during mind-wandering and self-referential thought. In complex environments, dynamically updating rich perceptual information can inform mental simulations. Accumulation of perceptual information during decision-making has been linked to the intraparietal sulcus in humans [46–49], in line with previous animal work [review: 50]. This region has also been implicated in executive control [51–53], risk-taking [54] and response inhibition [55], suggesting a multifaceted role in decision-making.

In the present study, we assessed fear generalization and avoidance under approach-avoidance conflict with functional magnetic resonance imaging (fMRI) in a transdiagnostic sample of adults with ARDs and healthy comparison participants (HC). To elucidate the heterogeneity of avoidance in ARDs, we examined effects of dimensional anxiety-related traits: anxiety sensitivity and intolerance of uncertainty, Anxiety sensitivity (i.e., “fear of fear”) is defined as the belief that anxiety-related sensations have harmful consequences, whereas intolerance of uncertainty (i.e., “fear of the unknown”) involves negative reactivity to uncertain situations and events. AS and IU have been identified as related but independent constructs relating to fear and anxiety [56], and converging evidence indicates that both are robust, transdiagnostic predictors of ARDs. Meta-analyses have identified elevated AS across anxiety disorders, OCD, and PTSD, with strongest associations found in panic disorder, GAD, and PTSD [57, 58]. IU is also elevated across anxiety disorders, OCD [59–61] and PTSD [62, 63]; with some meta-analytic evidence for a particularly strong relationship with GAD [60, 61]. In light of this evidence, transdiagnostic conceptualizations of ARDs increasingly evoke AS and IU [e.g., 64, 65] as core constructs. Relevant to the current study, AS and IU have previously been shown to heighten maladaptive generalized avoidance in a community sample [66]. However, the extent to which AS and IU might differentially contribute to avoidance behavior in ARDs remains unknown.

We applied a validated avoidance paradigm [67] to elicit decision-making in which avoidance is maladaptive by virtue of being both costly to task performance and unnecessary for safety [67, 68]. Neural activity and avoidance were assessed in response to true threat cues paired with shock, safety cues never paired with shock, and safe generalization stimuli with varying resemblance to the threat cue.

We first examined neural threat reactivity to task stimuli. We hypothesized that 1) threat reactivity in anterior insula and dmPFC would increase with threat-relevance of stimuli, and activity in vmPFC would show the opposite pattern, in line with previously-identified neural correlates of fear excitation and inhibition [29, 30, 34]. Then, we examined the relationship between Pavlovian fear and avoidance. We hypothesized that 2) Pavlovian activity in anterior insula and dmPFC would correlate with avoidance, and 3) generalized vmPFC activity would weaken Pavlovian-instrumental covariation, potentially reflecting adaptive inhibition of fear by the vmPFC. In line with prior findings [66, 69], we also hypothesized that 4) ARD diagnosis, anxiety sensitivity, and intolerance of uncertainty would strengthen Pavlovian-instrumental covariation.

We next examined neural activity during mental simulation in the task, applying multi-voxel pattern analysis (MVPA) to identify the accuracy with which mental simulation related brain activity could predict task behavior. We hypothesized that 5) neural activity corresponding to ‘imagine-approach’ in fear-related regions (anterior insula, dmPFC, vmPFC) would predict avoidance, and that this predictive strength would correlate with anxiety sensitivity, indicating a greater likelihood of avoidance in the context of fearful activation while imagining approach. We also hypothesized that 6) neural activity corresponding to both ‘imagine-approach’ and ‘imagine-avoid’ in hippocampus, PCC, vmPFC, and IPL would predict avoidance, and that this predictive strength would be negatively correlated with intolerance of uncertainty, reflecting less concordance of behavior with mental simulation of uncertain outcomes.

## Methods

### Participants

Participants were adults aged 18–60, with a current anxiety-related disorder diagnosis (ARD), or with no current psychiatric diagnosis (HC). Participants were included in the ARD group if they met criteria for a current diagnosis of an anxiety disorder, a trauma- and stressor-related disorder, or obsessive-compulsive disorder, consistent with prior ARD literature [2]. Those with current or past psychosis, current substance abuse, serious medical conditions that would interfere with participation, or MRI contraindications were excluded from participation. All participants gave written informed consent, and the experiment was approved by the institutional review boards of the University of Minnesota and the Minneapolis Veterans Affairs Health Care System (IRB #1701M03101, #1610M96641).

### Procedure

Following informed consent and clinical interviewing, eligible participants completed questionnaire measures. A shock workup procedure was then completed during which sample shocks were rated by participants to find a level that was moderately painful but tolerable, similar to previous studies [e.g., 70]. Participants then received instructions and task practice, entered the fMRI environment, and completed the avoidance task.

### Clinical measures

To determine ARD status, trained assessors administered the Structured Clinical Interview for DSM-IV [71] and Clinician-Administered PTSD Scale [72] to all participants. Diagnoses were assigned via consensus among at least three assessors. Participants also completed the Anxiety Sensitivity Index [73] and Intolerance of Uncertainty Scale [74].

### Avoidance task

**Fig. 2:**
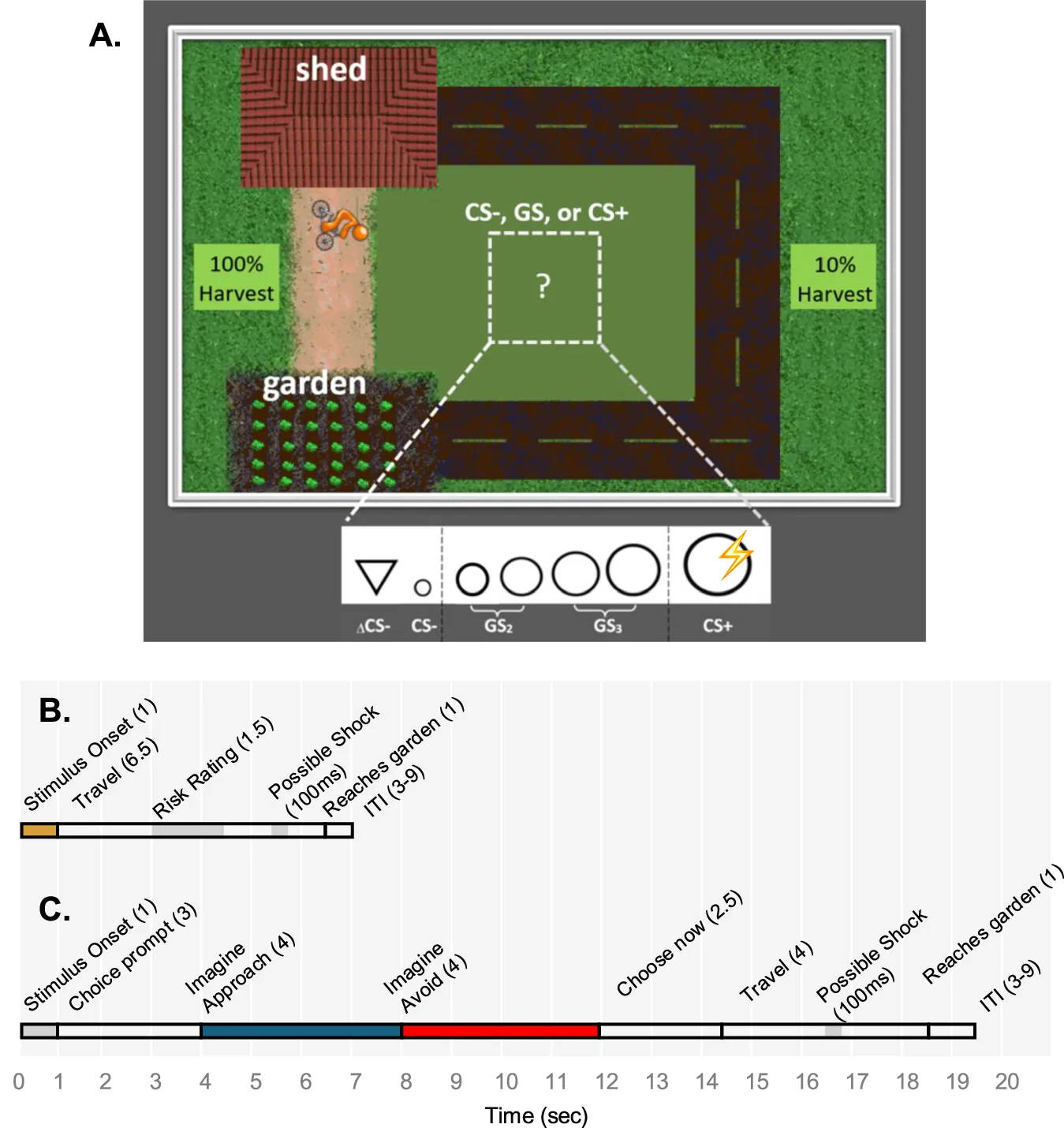
Avoidance task. **A** The task involved stimuli presented sequentially. Half of participants were presented the stimulus set displayed here, with the largest and smallest ring serving as CS+ and CS − , respectively. For the remaining half of participants this was reversed, with the largest and smallest rings serving as CS− and CS+, respectively. Generalization stimuli reflected a continuum of similarity between CS- and CS+; to limit the total length of the task, the two GSs closest in size to the CS- were omitted. Participants were instructed that the short road was dangerous (i.e., conferred risk of shock) but was associated with a high likelihood of successful harvest, whereas the long road was always safe but less likely to result in successful harvest. During 50% of CS+ trials, participants received an actual electric shock (unconditioned stimulus: US) together with a virtual US (images of the virtual farmer receiving a shock). On the other 50% of CS+ trials, participants received the virtual US but no actual US. **B** The task included passive trials to assess conditioned (i.e., Pavlovian) threat responses to stimuli. Following initial stimulus onset, participants passively viewed the farmer traveling the short road, rated their perceived risk of shock, and received the trial outcomes pertaining to shock and harvest. Neural activity corresponding to stimulus onset, shown in yellow, was assessed as an index of Pavlovian responding. **C** The task also included choice trials, to assess instrumental conditioning of avoidance behavior. Following stimulus onset, participants were prompted to imagine choosing each road, then were prompted to choose the long or short road, then received the trial outcomes. Neural activity corresponding to mental simulation, shown in red and blue, was assessed. Duration in seconds is shown after each trial event. Example trial timings are shown here; durations were jittered for stimulus onset (1–2 sec), travel time (3–6.5 sec), and inter-trial intervals (3–9 sec), and the order of “imagine approach” and “imagine avoid” was counterbalanced across instrumental trials. ΔCS − = triangular conditioned safety cue; oCS − = ring shaped conditioned safety cue; GS_2_ and GS_3_, = generalization stimulus classes 2 and 3; CS+ = conditioned danger-cue; ITI: inter-trial interval.

The avoidance task took the form of a computer game in which a farmer traveled from a shed to a garden to harvest crops, via a short road or a long road (Fig. 2A). On passive trials, the farmer automatically traveled down the short road; on choice trials, the participant could choose between the short and long roads. Participants were instructed that traveling the short road was dangerous (contingently associated with electric shock) but increased the likelihood of a successful harvest. Conversely, traveling the long road was never associated with shock but reduced the likelihood of successful harvest (see Supplement for details). The task was thus designed to induce approach-avoidance conflict, such that the motivation to avoid shock by taking the long road would compete with the motivation to gain a successful harvest by taking the short road.

#### Stimuli

Shock delivery while traveling the short road depended on the shape presented in the center of the screen. These shapes consisted of a triangle and circles of different sizes (see Fig. 2A), constituting the conditioned and generalization stimuli.

Circular stimuli included six rings of gradually increasing size, with extremes serving as conditioned danger (CS+) and conditioned safety (CS-) cues. The six rings of intermediary size were generalization stimuli (GSs) selected from a set of eight rings forming a continuum-of-similarity between CS+ and CS-; the two rings most similar to the CS- were omitted from the present study to limit the total duration of the experiment while ensuring adequate presentation of each stimulus-class for fMRI analysis. As has been done previously [66, 68, 75], responses to adjacent GS sizes were averaged for the present analysis, yielding two classes of GSs (GS_2_, GS_3_). CS+ size was counterbalanced, with the largest ring serving as CS+ for approximately half of participants, and the smallest ring serving as CS+ for the remaining participants. A triangle served as an outlier conditioned safety cue (ΔCS-). The unconditioned stimulus (US) was an electric shock delivered to the right ankle (2–5 mA, 100–200 ms, 1–3 sequential pulses) rated by participants as ‘moderately but not highly painful’.

#### Trial structure

Trials were either passive or choice trials. Trial structures are shown in Fig. 2B, C and described in detail in the Supplement.

##### Passive trials

Passive trials consisted of stimulus onset and a prompt to rate perceived risk of shock on a 3-point scale (“None”, “Some”, “A Lot”), followed by the farmer automatically traveling the short road. All CS+ passive trials involved a graphic of the farmer receiving a shock; the participant also received actual shock on 50% of CS+ trials. Partial reinforcement of the actual shock was chosen to attenuate habituation to repeated shocks, whereas full reinforcement of the virtual shock was used to maximize participants’ retention of the CS-US contingency, as has been done previously [e.g., 69].

##### Choice trials

Choice trials consisted of stimulus onset, followed by two mental simulation prompts, presented in counterbalanced order across trials: “Imagine taking the short road” (i.e., imagine-approach) and “Imagine taking the long road” (i.e., imagine-avoid). Participants were then prompted to choose a road, and finally observed the farmer traveling and reaching the garden, with the trial outcome involving shock and harvest. Choosing the short road on CS+ trials always resulted in the shock graphic as well as an actual shock, delivered 1–2.5 s after the farmer began traveling. Choosing the long road on CS+ trials never resulted in shock. No shocks were given for other stimulus-types on the short or long road.

#### Experimental phases

The paradigm consisted of an Acquisition phase followed by a Generalization phase (see Supplement for details). Acquisition includes 24 passive trials with 8 trials per stimulus type (ΔCS-, oCS-, CS+), all presented within one 6-minute run. Generalization consisted of 90 passive and 90 choice trials, with 18 passive and 18 choice trials for each stimulus type (ΔCS-, oCS-, GS_2_, GS_3_, CS+) presented across six, 9-minute runs.

### Data analysis

Data and code are available upon request.

#### Neural substrates of threat reactivity

We defined neural substrates of threat reactivity (i.e., Pavlovian conditioned fear) using whole-brain t-test comparisons of activation to the 1–2 s following stimulus onset to CS+ vs. ΔCS- were conducted via AFNI’s *3dttest*+*+* program. Clusters were set with clusterwise *p* < 0.05 and voxelwise *p* ≤ 5 × 10^−12^, using first nearest-neighbor clustering (faces touch), with a minimum cluster size of 50 voxels, based on nonparametric permutation testing using *3dclustsim*. We compared the resulting clusters with a priori hypothesized neural substrates of conditioned fear: dorsal anterior cingulate cortex (dACC), dorsomedial prefrontal cortex (dmPFC), bilateral anterior insula (AI), and ventromedial prefrontal cortex (vmPFC). Clusters that corresponded with a priori regions of interest were selected for analysis.

#### Task responding across stimuli

Task variables of interest included, for each stimulus-type, a) average risk ratings across trials, b) blood oxygen-level dependent (BOLD) signal corresponding to the 1–2 s period following stimulus onset (i.e., average beta across voxels within each region of interest), and c) avoidance behavior, defined as the proportion of choice trials on which the long road was chosen.

In line with prior work using this task [76–78], we assessed task responding across stimulus-types using linear mixed-effects regressions, with the task measure as the dependent variable and stimulus-type entered as an ordered factor and nested within subjects. We assessed effects of ARD diagnosis by including diagnostic status as a between-subject predictor; in separate models, anxiety sensitivity and intolerance of uncertainty were included as continuous between-subjects predictors; e.g., *Avoidance ~ Stimulus* × *Anxiety sensitivity*.

#### Pavlovian-instrumental covariation

To assess the degree of generalization for each participant, we computed participant-level generalization scores for each behavioral and neural response, defined as averaged responding across GSs with ΔCS- responses subtracted as an index of baseline responding (i.e., not reflecting fear generalized to all rings). We assessed correlations of generalization scores for Pavlovian responses (threat expectancy and neural threat reactivity) with generalization scores for avoidance, i.e., Pavlovian-instrumental covariation for generalization.

To examine anxiety-related individual differences, regression analyses were conducted assessing anxiety-related moderators of the effect of generalized Pavlovian responses (i.e., perceived risk or neural activity) on avoidance. ARD status, anxiety sensitivity, and intolerance of uncertainty were entered as moderators in separate models; e.g., *Avoidance*_*GS*_ *~ Threat expectancy*_*GS*_ × *Anxiety sensitivity*. To assess anxiety-related differences in adaptive avoidance to the true threat cue, we conducted similar regressions using CS+ responses, with ΔCS- responses subtracted; e.g., *Avoidance*_*CS+*_ *~ Threat expectancy*_*CS+*_ × *Anxiety sensitivity*.

Significant interaction effects were followed by calculations of simple slopes to illustrate the effect of the predictor variable (Pavlovian responses) on the outcome variable (avoidance) at low (−1 SD), moderate (mean), and high (+1 SD) values of the moderator (anxiety-related traits), or in ARD and HC separately. A significance threshold of α = 0.05 was applied.

#### Predicting avoidance with mental simulation related neural activity

##### Trial-level estimates

For imagine-approach and imagine-avoid separately, trial-level estimates of brain activity were computed with a GLM regression via AFNI’s @3dDeconvolve program, followed by AFNI’s @3dLSS program, in accordance with previous findings that this method yields trial-level estimates with high classification accuracy and signal-to-noise ratio for fast event-related designs [79].

##### Temporal feature selection

First, trial-level neural responses to mental simulation periods were entered as inputs to multi-voxel pattern analysis (MVPA). Brain areas yielding high accuracy from this analysis are conceptualized to represent brain processes occurring at any time during the deliberation period that are predictive of choice, e.g., mental simulation, conflict processing, or motor preparation. Analyses were repeated with imagine-approach, imagine-avoid, and both prompts together.

##### Classification

For each subject, each voxel in the brain was set as the center of a 5x5x5-voxel ‘searchlight’ [80, 81]. Trial-level betas within the searchlight were entered into a naïve Bayesian linear discriminant analysis with six-fold cross-validation: a training set, which consisted of brain responses and actual choices corresponding to five of the six task blocks (i.e., 75 of the 90 choice trials), was used to generate predicted choices for the test set, which consisted of the remaining task block, and this was repeated until each task block had served as the test set. This yielded a set of 90 predicted choices for each voxel, which were then compared with actual choices to yield a ‘hit rate’, or the proportion trials where avoidance was predicted and did occur, and a ‘false positive rate’, or the proportion of trials where avoidance was predicted and did not occur, which in turn yielded a detectability index, *d’*, for each voxel [82], reflecting the extent to which the searchlight centered on that voxel can accurately detect a subsequent avoidance choice. This analysis therefore yielded subject-specific brain maps reflecting which areas of the brain are most predictive of choice for a given subject.

##### Group analysis and individual differences

To identify which brain regions were generally predictive of choice across subjects, one-sample *t-*tests were conducted across all subjects’ volumes of *d’* results, and clusters were computed at a voxelwise alpha level of 0.005 using MATLAB’s *bwconncomp*. Following identification of each choice-predictive ROI, neural responses within the ROI preceding approach choices and avoidance choices were compared to guide interpretation of accuracy findings. We computed subject-level peak predictive accuracy within each cluster. We conducted *t* tests to compare accuracy in ARD vs. HC, and computed Pearson correlations with anxiety sensitivity and intolerance of uncertainty.

## Results

### Participant characteristics

Of the 153 participants who completed the task, 7 did not report conditioned CS+ threat expectancy (see Supplement) and were excluded. Additionally, 12 participants had excessive head motion during fMRI and 6 participants had other fMRI quality issues, leaving a sample of *n* = 135 subjects for fMRI analyses (see Supplement for power analysis). The ARD group was younger than HC and reported higher AS and IU; self-reported sex and race did not significantly differ across groups (Table 1).

**Table 1:** Demographic and Clinical Characteristics.

	Anxiety-Related Disorders (ARD; *n* = 58)	Healthy Comparison (HC; *n* = 77)	
	***M (SD)***	***M (SD)***	***p***
Age	32.78 (10.51)	37.18 (12.70)	0.034*
	***N (%)***	***N (%)***	
Sex			0.331
Female	26 (44.8%)	27 (35.1%)	
Male	32 (55.1%)	50 (64.9%)	
Race			0.529 †
Asian	4 (6.9%)	6 (7.8%)	
Black	2 (3.4%)	4 (5.2%)	
Multiracial	1 (1.7%)	0 (0.0%)	
White	48 (82.8%)	64 (83.1%)	
None of these	2 (3.4%)	0 (0.0%)	
Not reported	1 (1.7%)	3 (3.9%)	
	***M (SD)***	***M (SD)***	
Anxiety Sensitivity	23.02 (11.69)	12.41 (9.02)	<0.001***
Intolerance of Uncertainty	34.67 (8.24)	26.23 (7.93)	<0.001***
**Current Diagnoses**	***N (%)***		
Generalized anxiety disorder	24 (41.4%)	–	
Social anxiety disorder	20 (34.5%)	–	
Specific phobia	8 (13.8%)	–	
Anxiety disorder, not otherwise specified	8 (13.8%)	–	
Panic disorder	3 (5.20%)	–	
Agoraphobia w/o panic disorder	1 (1.70%)	–	
Posttraumatic stress disorder	15 (25.9%)	–	
Adjustment disorder	1 (1.70%)	–	
Obsessive-compulsive disorder	6 (10.3%)	–	
Comorbid Major depressive disorder	14 (24.1%)	–	
Comorbid Dysthymia	6 (10.3%)	–	

Anxiety-related disorders defined as anxiety disorders, trauma- and stressor-related disorders, and obsessive-compulsive disorder. Percentages do not add to 100 because some participants had more than one diagnosis.

*p < 0.05, ***p < 0.001 † denotes Fisher’s exact test.

### Pavlovian and instrumental conditioning

#### Threat expectancy

**Fig. 3:**
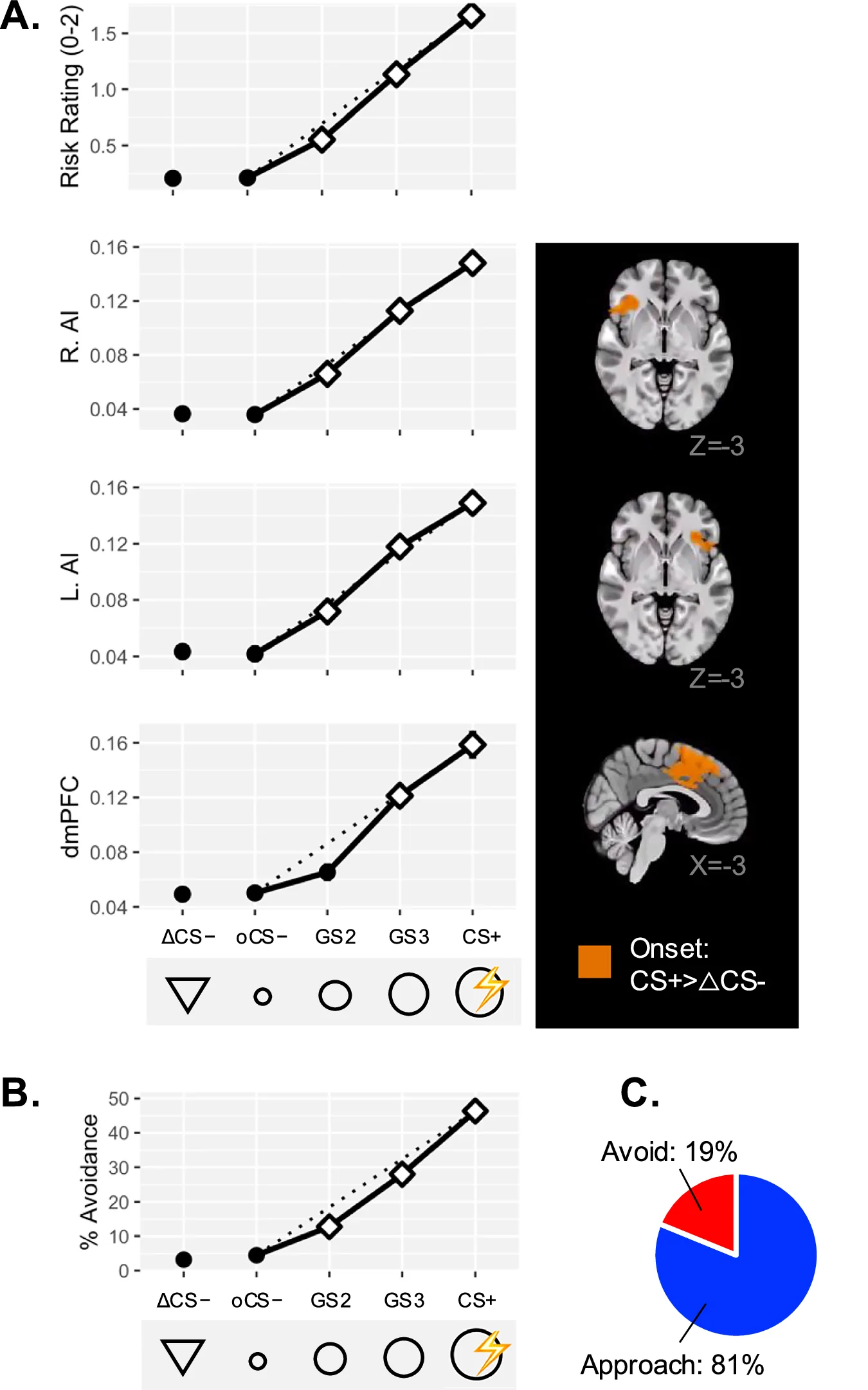
Conditioned fear and avoidance generalize from a true threat cue to safe stimuli. **A** Following initial stimulus onset, participants were asked to rate their perceived risk of shock (i.e., threat expectancy) following stimulus onset throughout the task. As expected, these risk ratings were lowest to safety cues and highest to the threat-cue (CS+), and gradually increased as safe stimuli increased in similarity to the CS+. Clusters of brain activity corresponding to threat reactivity with significant CS+ vs. ΔCS- contrast are shown, with corresponding activity to each stimulus-type. Neural threat reactivity in left anterior insula, right anterior insula, and dorsomedial prefrontal cortex similarly generalized from CS+ across safe stimuli resembling the CS+. **B** Participants were given the option to choose approach or avoidance on choice (i.e., instrumental) trials. The rate of avoidance generalized from CS+ across safe GSs. Bolded diamonds reflect significant *post hoc t-*test comparisons with ΔCS−, indicating the degree of generalization. **C** Across all participants, avoidance was chosen on 19% of instrumental trials. Error bars show standard error. ΔCS− = triangular conditioned safety cue; oCS− = ring shaped conditioned safety cue; GS = generalization stimulus; GS_2_ and GS_3_, = GS classes 2 and 3; CS+ = conditioned danger-cue; L. AI: left anterior insula; dmPFC: dorsomedial prefrontal cortex.

First, we assessed conditioned threat expectancy (i.e., risk ratings) to the CS+ and generalization across safe stimuli resembling the CS+. In both task phases, risk ratings to the CS+ were elevated relative to oCS- and ΔCS-, indicating expected conditioning effects (Table S1). As expected, in the generalization phase, threat expectancy gradually declined as the presented stimulus differentiated from CS+ (Fig. 3, Table S3), indicating generalization of threat-expectancy from the true threat cue across safe stimuli resembling the threat cue.

#### Neural threat reactivity

To assess neural indices of threat reactivity (i.e., Pavlovian conditioned fear), we next examined neural activity corresponding to the 1–2 s period following stimulus onset, in clusters with significant CS+ vs. ΔCS- contrast. Clusters overlapping with three of our a priori regions of interest were identified: left and right anterior insula and dmPFC (Table S2). As expected, threat reactivity in these clusters was elevated to CS+ and gradually decreased as rings differentiated from CS+, indicating robust coding of Pavlovian generalization in these regions. Contrary to expectations, activity in vmPFC did not show significant CS+ vs. oCS- contrast. Exploratory analyses with a separately-defined vmPFC cluster are detailed in the Supplement.

#### Avoidance

Analyses of avoidance behavior (i.e., instrumental conditioning) indicated that, as expected, avoidance behavior was highest to CS+, and gradually declined as stimuli differentiated from CS+ (Fig. 3, Table S3).

### Pavlovian-instrumental covariation

We next examined threat expectancy and neural threat reactivity as potential predictors of avoidance behavior (i.e., Pavlovian-instrumental covariation). Consistent with our hypotheses, generalization of threat expectancy was correlated with generalization of avoidance (*r* = 0.42, *p* < 0.001), as was generalization of dmPFC activity (*r* = 0.26, *p* = 0.002), in line with the putative role of dmPFC in fear expression. By contrast, right and left anterior insula activation to GSs were not significantly correlated with generalized avoidance (*p*s < 0.134). When examining responses to the true threat cue, threat expectancy and neural threat reactivity in all three regions of interest were correlated with CS+ avoidance, suggesting that adaptive fear prompted adaptive avoidance of true threat. (*r*s > 0.21, *p*s < 0.013).

### Anxiety-related differences in threat reactivity and avoidance

#### Effects of anxiety disorder diagnosis

We first examined the extent to which those with ARDs differed from HC in overall Pavlovian and avoidance responses to stimuli. Participants with ARDs showed more generalization of avoidance than HC, characterized by less CS+ avoidance and less-steep declines from CS+ across GSs (see Supplement).

**Fig. 4:**
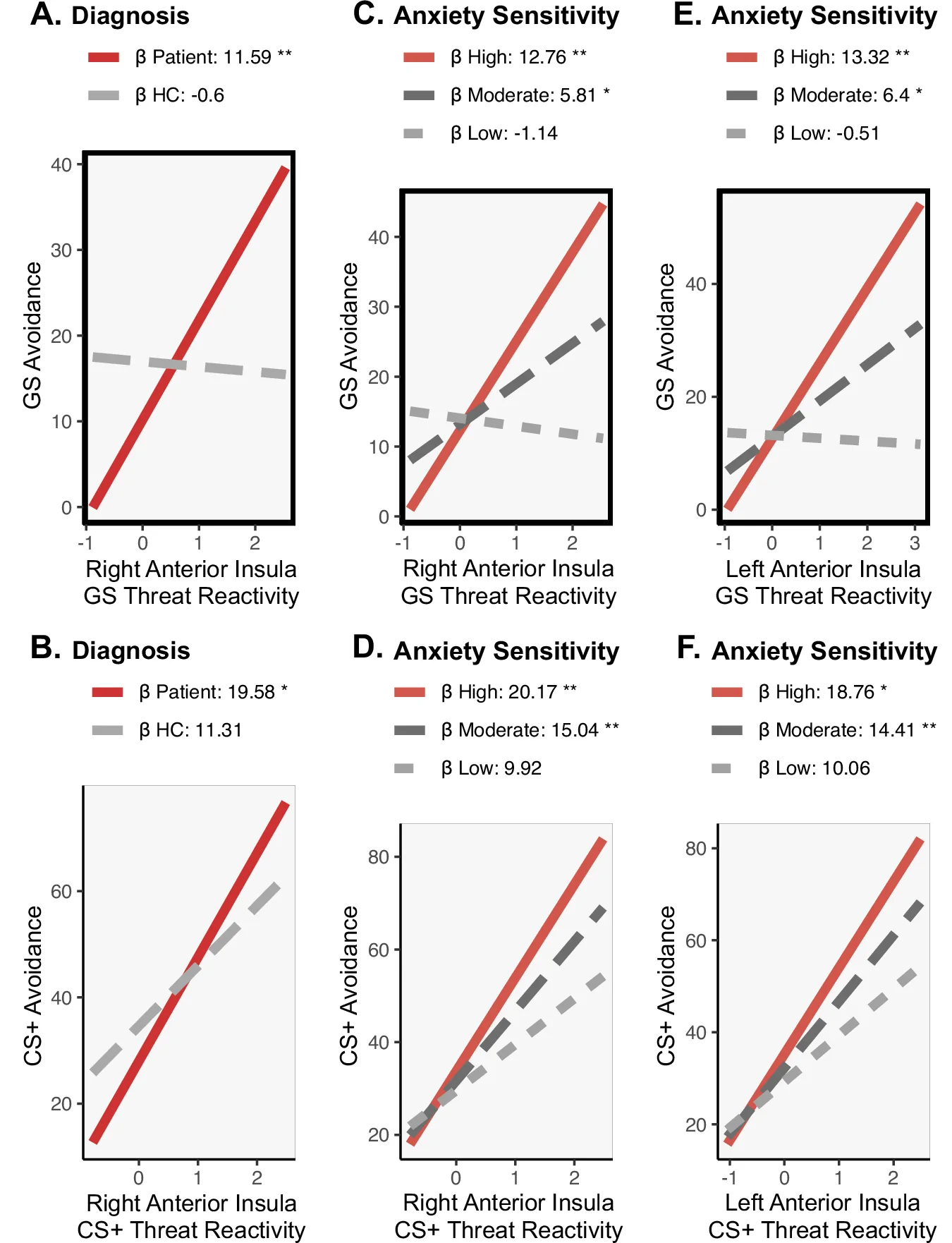
Anxiety sensitivity strengthens the link between initial threat reactivity and maladaptive avoidance. Models were conducted examining the moderating effect of anxiety-related disorders (ARDs) and anxiety sensitivity on Pavlovian-instrumental covariation: the link between initial (Pavlovian) threat reactivity and instrumental avoidance behavior (example model: *Avoidance ~ Group (ARD, HC)* × *Threat Reactivity)*. Results of simple slopes analyses are shown above each plot. Each plot shows threat reactivity on the *x*-axis and avoidance behavior on the *y*-axis. The top row of plots (A, C, E) shows results for safe generalization stimuli, which resembled the threat-cue but were never paired with shock. Slopes therefore represent the relationship between threat reactivity and unnecessary avoidance in safe situations. The bottom row of plots (B, D, F). shows corresponding results for true threat cues, such that slopes represent the relationship between threat reactivity and adaptive avoidance of true threat. **A** A significant positive relationship between generalized right anterior insula activity and maladaptive avoidance was seen among the ARD group only, suggesting ARD-related heightening of Pavlovian-instrumental covariation. **B** The relationship between right anterior insula reactivity to true threat cues and adaptive avoidance did not significantly differ across groups. **C** Next, effects of anxiety sensitivity across the sample were examined. Anxiety sensitivity scores were examined continuously in main analyses, simple slopes effects for high, moderate, and low levels of anxiety sensitivity are shown here for illustration purposes. Echoing patient vs. control findings, anxiety sensitivity moderated the relationship between generalized threat reactivity in right anterior insula and maladaptive avoidance, **D** but not the relationship between true threat reactivity and adaptive avoidance. **E, F** A similar pattern was observed in the left anterior insula. Black border indicates significant moderating effect of ARDs or anxiety sensitivity on Pavlovian-instrumental covariation, *p* < 0.05. Full moderation results are shown in Tables S10-A11. Corresponding ΔCS- values were subtracted from all GS_avg_ values. * *p* < 0.05, ** *p* < 0.01, *** *p* < 0.001. GS: safe generalization stimuli; CS+ : true threat cue.

Next, we examined moderating effects of diagnostic status on Pavlovian-instrumental covariation, to test the hypothesis that generalized threat reactivity would more readily potentiate maladaptive, generalized avoidance in those with ARDs compared to HC. Consistent with this hypothesis, a significant moderating effect of diagnostic status was found on the relationship between generalized right anterior insula activity and generalized avoidance (β = 8.61 [95%CI: 1.12 – 16.1], *p* = 0.025, η_p_^2^ =0.04), indicating that in the context of generalized threat reactivity in the anterior insula, those with ARDs were more likely to engage in maladaptive avoidance (Fig. 4A). When examining responses to the true threat cue, ARD diagnosis did not significantly moderate the relationship between right anterior insula and avoidance (*p* = 0.456; Fig. 4B). These findings support the conceptualization that those with ARDs engage in excessive maladaptive avoidance in safe situations, whereas adaptive avoidance in the context of true threat is common across those with ARD and HC. Other Pavlovian-instrumental relationships did not vary between those with ARDs and HC. (*p*s > 0.126, Table S10).

#### Effects of anxiety sensitivity and intolerance of uncertainty

We then examined the effects of two transdiagnostic anxiety-related traits – anxiety sensitivity and intolerance of uncertainty – on task responding. When examining overall responding to task stimuli, neither anxiety sensitivity nor intolerance of uncertainty were associated with differences in generalization of threat expectancy, neural threat reactivity, or avoidance (see Supplement). When examining Pavlovian-instrumental covariation, anxiety sensitivity moderated the effect of generalized right (β = 12.42 [2.02 – 22.81], *p* = 0.020, η_p_^2^ =0.04) and left anterior insula (β = 12.35 [1.08 – 23.62, *p* = 0.032, η_p_^2^ =0.03) activity on generalized avoidance. *Post hoc* simple slopes analyses indicated that Pavlovian-instrumental covariation strengthened with increasing anxiety sensitivity, supporting the hypothesis that threat reactivity would more strongly induce avoidance among those with greater “fear of fear”. By contrast, anxiety sensitivity did not significantly moderate Pavlovian-instrumental covariation for the true threat cue (*p*s > 0.342; Table S11, Fig. 4C–F), suggesting that effects of anxiety sensitivity are specific to maladaptive avoidance. Anxiety sensitivity did not significantly moderate Pavlovian-instrumental covariation for any other task indices, and intolerance of uncertainty did not moderate Pavlovian-instrumental covariation (Table S12).

### Decoding avoidance behavior from neural activity during mental simulation

We next examined neural activity during the mental simulation period immediately preceding the approach/avoidance decision. The neural correlates of “imagine-approach” and “imagine-avoid” prompts were largely overlapping (Figure S4), suggesting that neural activity during the two prompts could reflect general task engagement or action preparation. In MVPA analyses, approach versus avoidance choices could be predicted with high sensitivity using neural activity in superior parietal lobule (sample mean *d’*>0.80), left motor cortex (*d’*>0.78), and medial occipital cortex (*d’*>0.98) during imagine-approach, imagine-avoid, and across the entire mental simulation period. In the imagine-approach period, neural activity in the intraparietal sulcus (*d’*=0.78) and dmPFC (*d’*=0.77) could also predict choices, and when imagine-approach and imagine-avoid periods were combined (i.e., entire mental simulation period), neural activity in the left frontal pole (*d’*=0.84) could predict choices. Post-hoc examination of neural activity in these regions indicated greater activity preceding an avoidance choice than an approach choice. (Table S13, Figure S5).

### Anxiety-related differences in prediction of avoidance

**Fig. 5:**
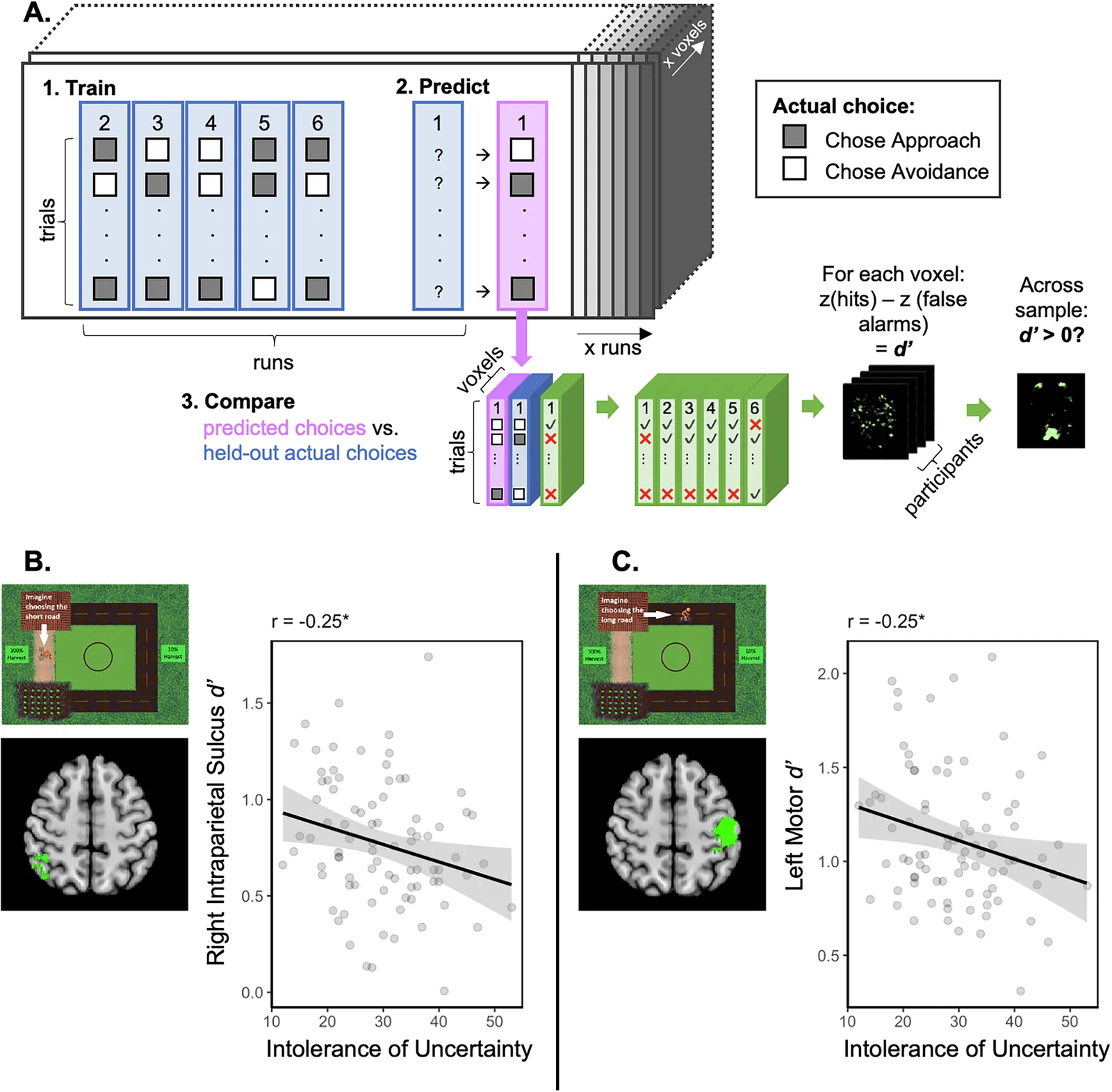
Neural activity during mental simulation predicts avoidance. **A** We entered neural activity corresponding to mental simulation (“imagine-approach” and “imagine-avoid”) into multi-voxel pattern analysis (MVPA) with unsupervised six-fold cross-validation, to predict trial-level approach (gray square) vs. avoidance (white square) choices. In 5x5x5-voxel ‘searchlights’ across the brain, for each of 6 task runs, the model was trained on the remaining 5 runs. Predicted choices (violet) were compared with actual choices (blue), to yield *d’* values indicating predictive accuracy. To identify brain regions predictive of avoidance across the sample, group-level clusters with significant predictive accuracy were determined with voxelwise threshold of α=0.005. Next, to assess individual differences in predictive accuracy, we conducted Pearson correlations between participant-level predictive accuracy and intolerance of uncertainty scores. Greater intolerance of uncertainty was associated with **B** lower peak predictive accuracy within right intraparietal sulcus  activity during “imagine-approach” (shown in inset) and **C** lower peak predictive accuracy within left M1 activity during “imagine-avoid” (shown in inset). *p < 0.05.

For those with greater intolerance of uncertainty, activity in the left motor cortex during the imagine-avoid period, and activity in the intraparietal sulcus during the imagine-approach period, were less predictive of choice (Fig. 5). Neither anxiety sensitivity nor ARD status were associated with differences in predictive accuracy (Table S13).

## Discussion

We investigated the transdiagnostic neural underpinnings of avoidance behavior in adults with anxiety-related disorders (ARDs), with a focus on a) initial threat reactivity and b) mental simulation. Threat reactivity in bilateral anterior insula and dorsomedial prefrontal cortex (dmPFC) generalized across safe stimuli resembling the threat cue, in line with our hypotheses and previous meta-analysis [30]. Neural activity during mental simulation, in a cortical network including intraparietal sulcus, dmPFC, and motor cortex, predicted avoidance behavior. Two novel findings regarding anxiety-related differences in avoidance emerged. First, those with ARDs and those with elevated anxiety sensitivity showed a stronger link between generalized threat reactivity in anterior insula and generalized, maladaptive avoidance. Second, those with greater intolerance of uncertainty showed weaker concordance of avoidance behavior with neural activity during mental simulation in intraparietal sulcus and motor cortex.

The finding of a stronger link between neural threat reactivity and avoidance (i.e., stronger Pavlovian-instrumental covariation) among those with elevated anxiety sensitivity echoes our prior work indicating a moderating role of anxiety sensitivity on relations between generalized fear-potentiated startle and generalized avoidance [66]. In another study, anxiety sensitivity similarly strengthened the relationship between fear of spiders and spider avoidance in youth [83]. In light of the known role of the anterior insula in responding to motivationally salient stimuli, particularly in the context of aversive threat-salience [29, 30], our present findings implicate threat reactivity in the anterior insula as a key driver of avoidance behavior. Together, these findings implicate heightened Pavlovian-instrumental covariation as a potential mechanism by which anxiety sensitivity increases risk for maladaptive avoidance, and in turn, ARDs [57, 84]. In contrast, intolerance of uncertainty was not found to moderate Pavlovian-instrumental covariation in the present study, contrary to our prior work in a community sample [66], suggesting that this prior finding might not extend to those with ARDs.

It is noteworthy that, unlike in the present findings, prior work has identified ARD-related overgeneralization of threat expectancy, fear-potentiated startle, and neural activity in passive viewing tasks without an avoidance option [e.g., 85, 86]. One interpretation is that in the present study, threat reactivity normalized over the larger number of trials included in the task compared to previous passive viewing paradigms. Another possibility is that the presence of an avoidance option attenuated threat reactivity in the present task. Specifically, despite the fact that Pavlovian trials were interspersed with instrumental trials during the generalization phase, the prospect of potential avoidance may have dampened initial threat reactivity to generalization stimuli among those with ARDs.

The present findings also diverge from prior literature in that results do not strongly support the role of the vmPFC in fear inhibition. Primary contrasts did not identify evidence of fear inhibition in the vmPFC, though effects in the expected direction did emerge at a less-stringent cluster significance threshold in secondary analyses. As noted above, the present task differs from traditional fear-conditioning tasks in that it includes a large number of trials and a decision-making component, both of which could have attenuated vmPFC effects. Furthermore, the vmPFC has been implicated in valuation and conflict signaling as well as fear inhibition [87, 88]; though some studies have identified distinct vmPFC subregions associated with these functions [89], functional overlap in the present sample could have made fear-inhibition signals difficult to detect. Using the vmPFC cluster obtained from secondary analyses, we did observe a moderating effect of vmPFC activity on the relationship between generalized fear excitation and avoidance, providing some support for the hypothesis that vmPFC activity lessens maladaptive avoidance.

When examining neural activity corresponding to mental simulation, our hypothesis that activity in regions associated with fear excitation and mental simulation would predict avoidance was partially supported. It is important to consider interpretation of our mental simulation findings in light of the observation that neural activity during “imagine-approach” and “imagine-avoid” simulation was largely overlapping (see Supplement). This overlap suggests that the decoded signal could include general task engagement, motor preparation, or conflict processing, instead of or in addition to distinct mental simulations of approach versus avoidance. “Imagine-approach” compared to “imagine-avoid” elicited greater activity in visual processing regions, suggesting greater overall attentional engagement while contemplating approach. Taken together with the observation that approach was chosen in 81% of trials overall, one interpretation is that participants often quickly decided to approach, and were more attentionally engaged during “imagine-approach”. For instance, when confident that a safety-cue was present, participants likely made a rapid decision to approach prior to the mental simulation period of the trial. Multi-voxel pattern analysis indicated that greater dmPFC and intraparietal sulcus activity during “imagine-approach” predicted avoidance behavior, suggesting that avoidance behavior arose from both a) bottom-up attentional orienting to threat and b) top-down executive control. Activity in vmPFC and hippocampus, putatively associated with mental simulation, did not predict approach or avoidance behavior.

Those with greater intolerance of uncertainty showed less concordance of avoidance behavior with mental simulation related neural activity, in line with our hypotheses. Specifically, for individuals with higher intolerance of uncertainty, avoidance was less accurately predicted by: a) activity during imagine-approach in intraparietal sulcus, a region associated with perceptual evidence accumulation and response inhibition [55, 90, 91], and b) activity during imagine-avoid in the motor area corresponding to the right hand [92], which was used to make decisions in the task. An important consideration in interpreting this finding is that the majority of decisions in the task were approach decisions. Dual models of action control propose a goal-directed system and a habit system for decision-making [93]; avoidance decisions in the present task can therefore be considered in terms of extent to which approach was habitual, and the extent to which avoidance was goal-directed. Thus, one interpretation is that individuals who find uncertainty more tolerable formed a strong approach habit, and avoidance only occurred after a concerted effort to evaluate evidence of threat, inhibit prepotent approach, and motorically prepare to avoid. By contrast, individuals with elevated intolerance of uncertainty may have had difficulty establishing and maintaining a prepotent approach habit; more readily changing their behavior in response to feelings of uncertainty, such that a wider variety of brain states preceded avoidance. This result mirrors prior findings that used a different approach-avoidance task to identify heightened decision uncertainty among individuals with mood and anxiety disorders [94, 95]. Taken together, these findings suggest that cognitive and behavioral arbitration of uncertainty may be a key target for intervention, particularly for those with elevated intolerance of uncertainty.

The divergent findings regarding anxiety sensitivity and intolerance of uncertainty shed light on potential heterogeneity of maladaptive avoidance behavior (see Fig. 1). Anxiety sensitivity (“fear of fear”) was associated with more avoidance in the context of initial threat reactivity, whereas intolerance of uncertainty (“fear of the unknown”) was associated with a weaker link between mental simulation related neural activity and avoidance. This distinction can guide future research on interventions for avoidance behavior. Patients with elevated anxiety sensitivity may benefit from interventions focused on fear reduction in safe situations (e.g., exposure-based therapies [96]). By contrast, the presence of elevated intolerance of uncertainty may indicate an intervention strategy focused on improving the efficiency of mental simulation when making decisions. Cognitive restructuring techniques, or behavioral techniques that focus on engaging in valued behaviors, such as behavioral activation [97] or acceptance and commitment based therapies [98], may be adaptable for this purpose. Future intervention studies are needed to test and refine these hypotheses, including laboratory studies testing methods for reducing avoidance in controlled behavioral paradigms, and clinical trials testing treatment strategies for reducing real-world avoidance.

## Limitations

Real-world avoidance is highly heterogeneous, and different forms of avoidance might vary in their neural correlates and their relationships with anxiety sensitivity and intolerance of uncertainty. The present findings should be therefore considered in light of limitations inherent in a) the characterization of real-world avoidance in the sample, and b) the experimental paradigm. Nuanced assessment of real-world avoidance symptoms, such as through experience sampling [99], is a promising avenue for future research to more closely link lab-based avoidance indices with real-world behavior patterns and improve the ecological validity of avoidance paradigms [100]. Relatedly, identification of disorder-specific avoidance patterns in the present sample is complicated by sample size and ARD comorbidity within the sample; future research with larger, “pure” diagnostic groups could identify disorder-specific neurobehavioral features. Regarding the experimental paradigm, it should be noted that our task elicited binary approach-avoidance decision-making in a controlled environment, whereas real-world avoidance can be graded, context dependent, and dynamically adjusted over time. Future research using paradigms that elicit graded avoidance [22, 101], escape [102], and other behaviors can assess the generalizability of the present findings.

## Conclusion

Taken together, these findings elucidate the neural basis of maladaptive avoidance in anxiety-related psychopathology. Enhanced coupling of generalized threat reactivity in the anterior insula with maladaptive avoidance is identified as a transdiagnostic neurobehavioral marker for ARDs. In an extension of prior work, this coupling was also found to strengthen with increasing anxiety sensitivity. By contrast, those with greater intolerance of uncertainty showed less concordance of avoidance with mental simulation related neural activity. Future work further refining dimensional models of maladaptive avoidance [12, 103], and testing neuroscience-informed interventions, holds promise to improve the conceptualization and treatment of anxiety-related psychopathology.

## Supplementary information

Supplement

## Data Availability

De-identified data and analysis code are available upon request.
