# Supplementary material for: Anxiety sensitivity and intolerance of uncertainty track distinct neurobehavioral dimensions of avoidance in anxiety-related disorders
Source: Mol Psychiatry. 2026 May 27;31(10):5561–73. doi: 10.1038/s41380-026-03640-1 (PMC13313569; doi:10.1038/s41380-026-03640-1)
Supplement: Supplementary file 1 [file 41380_2026_3640_MOESM1_ESM.docx]

**Anxiety sensitivity and intolerance of uncertainty track distinct neurobehavioral dimensions of avoidance in anxiety-related disorders**

**SUPPLEMENTARY MATERIALS**

**Supplemental Methods**

**Participants**

Recruitment was guided by power analyses indicating that a sample of at least *n*=124 would yield 80% power to detect small-to-medium effects (*f^2^*=.10) for moderation analyses examining interaction effects of Pavlovian threat reactivity and anxiety-related traits (e.g., anxiety sensitivity, intolerance of uncertainty) on avoidance behavior.

Acquisition of conditioned US expectancy was used as an inclusion criterion for the present analyses, such that all participants whose average risk rating to CS+ was less than their average risk rating to △CS- were excluded. Group comparisons indicated that the AD group was significantly younger than the HC group. Accordingly, primary analyses comparing patients to HC were repeated with age as a covariate, this did not substantially change results and is not reported here.

**Equipment**

Participants were given a 3-button fiber optic response pad (Lumina LP-404 by Cedrus, San Pedro, CA) to make risk ratings and avoidance decisions. Presentation software (Neurobehavioral Systems) was used to present the task and record risk ratings, avoidance decisions, and corresponding response latencies. The computer running Presentation interfaced with a PsyLab psychophysiology system that delivered shocks.

A 3T Siemens MAGNETOM Prisma with Tim 4G technology and a 32-channel parallel imaging head/neck coil was used to acquire functional T2*-weighted EPIs (TR=1500ms, TE=28ms, flip=90°, whole brain, 36 3.5mm axial slices, matrix=110x110, FOV=192cm) and high-resolution T1-weighted MP-RAGE sequences (224 1.0 mm sagittal slices; FOV=256 mm, ACQ=1, TR=2300ms, TE=3.6ms; matrix=256x256; TI=1100ms, bandwidth=180 HZ / pixel=46kHz for 256 pixels).

**Trial Structure**

Each Pavlovian and instrumental trial is followed by a jittered ITI of either 3, 5, 7 or 9 s with the frequency of each duration of ITI being 52%, 26%, 14%, and 8%, respectively. During ITIs, a farmer task graphic without images of the farmer or CS/GS is presented.

In tandem with shock delivery, the virtual farmer is graphically shown receiving a “virtual shock”. Additionally, on the 50% of CS+ Pavlovian trials not paired with “actual shock”, the farmer continues to be graphically shown receiving a virtual shock with the same time-course as actual shocks, in order to further reinforce the CS+/US association while limiting subjects’ habituation to the US.

***Pavlovian Trials***

To achieve jittered intervals between intra-trial events, the time-course of Pavlovian trials took 1 of 4 forms, with 25% of trials following each of four structures. When shocks are delivered, shock onset occurs at 5.5-6.5 s post-CS+ onset for trials with risk ratings, and at 3-4 s post-CS+ onset for CS+ trials without risk ratings. All Pavlovian trials begin with the presentation of a CS (_∆_CS-, CS-, CS+) or GS (GS_1_, GS_2_) which remains onscreen (in the center of the farmer task graphic) for the full 7-8.5 s duration of the trial. At 1 or 2 s post-trial onset, the farmer automatically begins traveling the short road. On half of trials, the question “Level of risk?” appears at the top of the screen 2 s into the farmer’s trip. This question cues participants to rate their perceived risk of shock on a 3-point scale, where 0 = “None”, 1 = “Some”, and 2 = “A Lot”. Participants are instructed to answer as quickly as possible with their index finger. Reaction times exceeding 2.5 standard deviations above the average are considered outliers and discarded (Ratcliff, 1993).

***Instrumental Trials***

For instrumental trials, each of four structures is applied to 25% of trials, to achieve jittered intervals and counterbalancing of intra-trial events. Trials begin with the presentation of a CS or GS in the center of the farmer task graphic (see Figure 1). The CS/GS remains onscreen for the duration of the trial which ranges between 20-21.5 s. At 1-2.5 s post- trial onset, the words “You will soon choose a road” appear at the top of the screen for 2-3.5 s, indicating an upcoming opportunity to choose between roads. Next, participants are given 8 s to imagine what it would be like to choose the short and long road (for separate analyses not described here). The words “Imagine choosing the long road” (4 s) and “Imagine choosing the short road” (4 s) are serially presented at the top of the screen along with a static visual of the farmer traveling the corresponding road. The order of short and long imaginal periods is counterbalanced across trial structures. At the start of the 8 s imaginal period, information on the probability of a successful harvest (i.e., probability of winning) for the short versus long road appears on the left and right side of the screen, respectively (see Figure 1), and remains on screen for the remaining duration of the trial. On 50% of trials for each CS/GS type, the probability of winning when choosing the short versus long road is 100% versus 10%. On the other half of trials, the chance of winning on the short versus long road is 75% versus 35%. Participants’ imagined outcomes are thus elicited in the presence of both the CS/GS and information on the differential likelihood of winning across the two roads. In the present analyses, all choice trials are combined across win-probabilities.

Following the imaginal period, the words “Which way?” appear at the top of the screen and participants are given 2.5 s to choose between sending the farmer down the short road or the long road. At the end of the 2.5 s choice window, the farmer is shown traveling the chosen road and reaching the garden 3-4 s thereafter.

**Experimental Phases**

During the Acquisition phase, to ensure an even distribution of trial types across acquisition, trials are randomly arranged in four blocks of six trials, with each block including 2 trials of each stimulus-type. Additionally, risk rating are assessed quasi randomly on 3 of 6 trials per block.

During the Generalization phase, instrumental trials include short/long winning probabilities of 100/10 or 75/35, with each set of probabilities applied to half of instrumental trials for each stimulus type. For each of six generalization runs, trials are arranged randomly in 3 blocks of 10 trials, with each Pavlovian and instrumental trial type presented once per block, and risk ratings probed on 2-3 Pavlovian trials per block. Additionally, the short/long winning probabilities for instrumental trials within a block are 100/10 for 2-3 trials and 75/35 for 2-3 trials (winning probabilities were varied for separate analyses not described here). Finally, Pavlovian and instrumental trials are randomly interleaved with the constraint that no more than two Pavlovian or instrumental trials occurred consecutively.

**fMRI Analysis**

Functional volumes were coregistered to the T1 volume and transformed to MNI space via AFNI's @SSwarper, subjected to standard pre-processing for slice-time correction and spatial smoothing with a 4 mm Gaussian blur, and scaled such that each voxel had a mean of 100. Individual-level functional activation maps were generated with a whole-brain general linear model (GLM) via AFNI’s 3dDeconvolve program, regressing the timecourse of each voxel’s blood oxygenation-level dependent (BOLD) signal during the generalization phase onto a gamma-variate ideal response function convolved with the timeseries of task events, including Pavlovian onset for each stimulus-type (△CS-, oCS-, GS2, GS3, CS+). Baseline drift, participant-specific movement (modeled with six motion parameters) and task events including shock delivery were entered as covariates of no interest.

**Identifying Neural Substrates of Choice Deliberation**

Neural substrates of choice deliberation were defined as brain regions demonstrating a significant response to the imaginal periods, based on t-test comparisons of beta weights for the imagine-approach period versus baseline and beta weights for the imagine-avoid period versus baseline, as well as an imagine-approach versus imagine-avoid contrast, via AFNI’s @3dttest++ program. Clusters were set with clusterwise p <0.05 and voxelwise *p* ≤ 5x10^-12^, using first nearest-neighbor clustering (faces touch), with a minimum cluster size of 50 voxels. These clusters were used to aid in interpretation of subsequent analyses.

**Supplemental Results**

**Pavlovian Acquisition**

To follow up results from the primary CS+ versus △CS- contrast, an additional CS+ versus △CS- contrast was conducted with a less-stringent threshold (*p*<.0002), to enable exploratory analyses of the vmPFC. This analysis yielded a large cluster spanning the vmPFC and dmPFC; voxels in the vmPFC were manually separated for exploratory analyses.

As expected, negative gradients in left vmPFC reflect strongest responding to △CS–, with decreases as stimuli increase in similarity to the CS+ (main effects of stimulus: linear: B=2.24[1.27—3.2], *p*<.001, *d*=.45; quadratic: B=1.45,[0.48—2.42, *p*=.004, *d*=.29). (Figure S1).

Anxiety-Related Differences in Pavlovian and Instrumental Generalization

There was a significant stimulus-type by AD diagnosis interaction on the linear component of the generalization gradient for avoidance, reflecting a steeper, more linear gradient among HCs (β=-34.33 [95% CI -38.55 – -30.12], *p*<.001, *d*=-2.07) than AD (β=-27.96 [95% CI -32.68 – -23.24], *p*<.001, *d*=-1.68). Greater rates of avoidance to CS+ were also observed among HC than AD, although the effect was not significant (*t*(144) = 1.29, *p* = .198, *d* = 0.21); groups did not differ in avoidance to other stimuli (*p*s>.24) (Table S5). These findings indicate that AD demonstrated blunted CS+ avoidance and greater generalization of avoidance relative to healthy comparison participants (Figure S2). No significant interactions of stimulus-type by AD diagnosis were seen on indices of Pavlovian generalization. (Table S5).

No significant effects of IU or AS, or their interactions with stimulus-type, were found (Tables S6-S7).

**Inhibitory Effects of vmPFC on Aversive Pavlovian-Instrumental Covariation**

Because CS+ responding is lower than CS- responding for the vmPFC, higher generalization scores reflect greater similarity of the GS response to the CS- response. Generalization of left vmPFC activity had a significant moderating effect on the relationship between generalization of left AI and generalization of avoidance, as well as the relationship between generalization of dmPFC and generalization of avoidance. In both cases, greater APIC-G was seen in individuals with *less* activation of left vmPFC to GSs, supporting the hypothesis that vmPFC activity lessens maladaptive avoidance (Figure S1). By contrast, APIC to true threat-cues (APIC-CS+) did not differ across levels of left vmPFC activity to CS+ for any of the indices measured (Table S9). These findings suggest that activations in left vmPFC, a putative fear inhibition region, may attenuate maladaptive APIC-G but not the more adaptive APIC-CS+.

Interactions of vmPFC, Fear Excitation Regions, and Anxiety on Avoidance

As an exploratory analysis examining the role of vmPFC in anxiety-related differences in APIC-G, regressions were conducted examining the three-way interaction of generalization of Pavlovian fear-excitation regions, generalization of vmPFC, and anxiety-related traits on generalized avoidance. Anxiety-related *weakening* of the effect of vmPFC on APIC-G would suggest that the vmPFC insufficiently blocks maladaptive avoidance in anxious individuals. By contrast, anxiety-related *heightening* of the effect of vmPFC on APIC-G would suggest that recruitment of fear-inhibitory regions could serve to effectively attenuate avoidance among anxious individuals.

When examining the three-way interaction effects of left vmPFC, diagnostic status, and indices of generalized fear excitation (i.e., generalization of anxiety ratings, risk ratings, left and right anterior insula, and dmPFC) on generalization of avoidance, the moderating effect of diagnostic status on APIC-G was not found to significantly differ across levels of generalization in left vmPFC, *p*s > 0.25.

The three-way interaction of generalization of left AI, generalization of left vmPFC, and anxiety sensitivity on generalization of avoidance was not significant (η_p_^2^ = .03, *p* = .069). However, given the small-to-medium effect size, an exploratory, follow-up simple slopes analysis was conducted to detect the direction of this effect. These follow-up analyses indicated that greater left AI activation to GSs was associated with more generalized avoidance in the context of 1) greater anxiety sensitivity and 2) lower left vmPFC activity to GSs. (Figure S3). This suggests that left vmPFC activity to generalization stimuli may be protective against APIC-G among individuals with elevated anxiety sensitivity. The moderating effect of anxiety sensitivity on APIC-G for other indices did not significantly differ across levels of generalization in left vmPFC, *p*s > .28.

The moderating effect of intolerance of uncertainty on APIC-G did not significantly differ across levels of generalization in left vmPFC, *p*s > 0.17.

Neural Substrates of Choice Deliberation

Imagine-avoid and imagine-approach periods elicited similar activation across trials (Figure S4), including activation of medial premotor cortex, frontoparietal regions, and posterior occipital cortex; and deactivation of the vmPFC, precuneus, and hippocampus. The imagine-approach prompt also elicited activations of bilateral anterior insula and thalamus, likely reflecting the higher reward-salience associated with the short path on the majority of trials (although the reward-salience of winning may have competed with the reward-salience of safety from shock on risky trials), together with the higher threat-salience associated with the short path on risky trials. When contrasting imagine-avoid with imagine-approach, differences were seen in the medial occipital cortex along the right calcarine sulcus.

**Table S1.** Conditioning of risk ratings and anxiety ratings

|  | Mean (SD) | | | | | | Median difference [95% CI] | | |
| --- | --- | --- | --- | --- | --- | --- | --- | --- | --- |
|  | tCS- | | oCS- | | CS+ | | CS+ vs. tCS- | | CS+ vs. oCS- |
| ACQ Anxiety Ratings (0-10) | 1.65 (2.02) | | 2.19 (2.27) | | 7.01 (2.46) | | 6.00 [5.00–6.50] *** | | 5.00 [5.00–6.00] *** |
| ACQ Risk Ratings (0-2) | 0.40 (0.49) | | 0.39 (0.48) | | 1.34 (0.40) | | 1.08 [1.00–1.25] *** | | 1.13 [1.00–1.25] *** |
| GEN Anxiety Ratings (0-10) | 0.78 (1.38) | 1.24 (1.87) | | 7.18 (2.31) | | 6.67 [6.33–7.00] *** | | 6.00 [5.33–6.67] *** | |
| GEN Risk Ratings (0-2) | 0.21 (0.33) | 0.21 (0.38) | | 1.66 (0.36) | | 1.56 [1.44–1.67] *** | | 1.56 [1.50–1.67] *** | |

Results of paired-sample sign test. tCS-: triangular safety-cue; oCS-: ring-shaped safety-cue; CS+: threat-cue. *** *p* < 0.001

**Table S2.** Coordinates of Pavlovian regions of interest.

| Label | #Voxels | CM X | CM Y | CM Z |
| --- | --- | --- | --- | --- |
|  | 2177 | 0.4 | 9.3 | 0.8 |
| **dmPFC** | **2163** | **-0.3** | **-10.5** | **44.7** |
|  | 1146 | -45.9 | -10.6 | 32.6 |
| **R AI** | **908** | **-38.2** | **-23.5** | **-1.3** |
| **L AI** | **751** | **34.8** | **-21.3** | **0.5** |
|  | 316 | 60.2 | 33.1 | 26 |
|  | 250 | -14.2 | 68.8 | 9.8 |
|  | 224 | 13.7 | 68.7 | 30.9 |
|  | 218 | 8.6 | 10.1 | 70.6 |
|  | 152 | 13.1 | 73.2 | 9.3 |
|  | 148 | -14.9 | 66.3 | 33.8 |
|  | 116 | -55.4 | 29.3 | 27.5 |
|  | 102 | 36.2 | 5.3 | 52.7 |
|  | 65 | -33.1 | 43.6 | 4.8 |
|  | 64 | -32.1 | 54 | 46.2 |
|  | 58 | 21.8 | 47.6 | 68.4 |
|  | 58 | 32.6 | 46.2 | 3.9 |
|  | 57 | 8.1 | 47.9 | 60.9 |
|  | 56 | 13.6 | 27.4 | 39.6 |

Rows in bold indicate clusters that overlapped with *a priori* regions of interest and were therefore included in the present analysis.

**Table S3. Pavlovian and Instrumental Generalization.**

|  | | β | 95% CI (Lower) | 95% CI (Upper) | *p* | η_p_^2^ |
| --- | --- | --- | --- | --- | --- | --- |
| Risk Rating | | 0.50 | 0.48 | 0.53 | <.001*** | .81 |
| R. Anterior Insula | | 3.84 | 3.48 | 4.19 | <.001*** | .53 |
| L. Anterior Insula | | 3.68 | 3.34 | 4.02 | <.001*** | .53 |
| dmPFC | | 3.81 | 3.41 | 4.21 | <.001*** | .46 |
| L. vmPFC | | -1.00 | -1.44 | -0.56 | <.001*** | .05 |
| Avoidance | 14.34 | | 142.87 | 15.80 | <.001*** | .48 |

RR: risk rating; R.: right; L.: left; dmPFC: dorsomedial prefrontal cortex.

Table S4. Correlations among Pavlovian indices and anxiety-related traits

|  | Gen.RR | Gen.R. AI | Gen.L. AI | Gen.dmPFC | Gen.L. vmPFC | CS+ RR | CS+ R. AI | CS+ L. AI | CS+ dmPFC | CS+ L. vmPFC | IU |
| --- | --- | --- | --- | --- | --- | --- | --- | --- | --- | --- | --- |
| Gen.RR |  |  |  |  |  |  |  |  |  |  |  |
| Gen.R. AI | 0.087 |  |  |  |  |  |  |  |  |  |  |
| Gen.L. AI | 0.108 | 0.884*** |  |  |  |  |  |  |  |  |  |
| Gen.dmPFC | 0.14 | 0.787*** | 0.776*** |  |  |  |  |  |  |  |  |
| Gen.L. vmPFC | -0.210* | 0.058 | 0.182* | 0.143 |  |  |  |  |  |  |  |
| CS+ RR | 0.501*** | 0.226** | 0.220* | 0.164 | -0.280*** |  |  |  |  |  |  |
| CS+ R. AI | -0.135 | 0.750*** | 0.678*** | 0.572*** | 0.037 | 0.222** |  |  |  |  |  |
| CS+ L. AI | -0.128 | 0.655*** | 0.732*** | 0.548*** | 0.1 | 0.224** | 0.915*** |  |  |  |  |
| CS+ dmPFC | -0.035 | 0.655*** | 0.641*** | 0.740*** | 0.013 | 0.260** | 0.827*** | 0.815*** |  |  |  |
| CS+ L. vmPFC | -0.253** | 0.180* | 0.261** | 0.119 | 0.700*** | -0.259** | 0.290*** | 0.345*** | 0.163 |  |  |
| IU | -0.001 | -0.073 | -0.095 | -0.054 | -0.095 | 0.066 | -0.016 | -0.008 | -0.016 | 0.02 |  |
| AS | 0.004 | -0.101 | -0.185* | -0.07 | -0.112 | 0.081 | -0.082 | -0.115 | -0.021 | -0.071 | 0.712*** |

Gen.: neural activity averaged across generalization stimuli; CS+: neural activity during threat-cue; R.: right; L.: left; AI: anterior insula; dmPFC: dorsomedial prefrontal cortex; vmPFC: ventromedial prefrontal cortex; RR: risk rating; IU: intolerance of uncertainty; AS: anxiety sensitivity. Neural or behavioral responses to the triangular safety-cue were subtracted from all Gen. and CS+ indices. **p*<.05; ** *p*<.01,; *** *p* < 0.001.

**Table S5. Effect of Anxiety-Related Disorder Status on Pavlovian and Instrumental Generalization**

|  | β | 95% CI (Lower) | 95% CI (Upper) | *p* | η_p_^2^ |
| --- | --- | --- | --- | --- | --- |
| RR | 0.03 | -0.02 | 0.08 | .187 | <.01 |
| R. AI | -0.13 | -0.85 | 0.59 | .731 | <.01 |
| L. AI | -0.23 | -0.92 | 0.45 | .506 | <.01 |
| dmPFC | -0.03 | -0.85 | 0.79 | .948 | <.01 |
| L. vmPFC | -0.67 | -1.55 | 0.21 | .136 | <.01 |
| Avoidance | -0.86 | -3.82 | 2.10 | .570 | <.01 |

RR: risk rating; R.: right; L.: left; AI: anterior insula; dmPFC: dorsomedial prefrontal cortex; vmPFC: ventromedial prefrontal cortex.

**Table S6. Effect of Anxiety Sensitivity on Pavlovian and Instrumental Generalization**

|  | β | 95% CI (Lower) | 95% CI (Upper) | *p* | η_p_^2^ |
| --- | --- | --- | --- | --- | --- |
| RR | 0.01 | -0.02 | 0.03 | .577 | <.01 |
| R. AI | -0.28 | -0.64 | 0.07 | .121 | <.01 |
| L. AI | -0.28 | -0.62 | 0.06 | .107 | <.01 |
| dmPFC | -0.07 | -0.48 | 0.34 | .728 | <.01 |
| L. vmPFC | -0.29 | -0.73 | 0.15 | .198 | <.01 |
| Avoidance | 1.26 | -0.21 | 2.73 | .094 | <.01 |

Stimulus * Anxiety sensitivity interaction effects are reported for each task measure, to indicate the effect of anxiety sensitivity on generalization slopes. Anxiety sensitivity scores are scaled and mean-centered. RR: risk rating; R.: right; L.: left; AI: anterior insula; dmPFC: dorsomedial prefrontal cortex; vmPFC: ventromedial prefrontal cortex.

**Table S7. Effect of Intolerance of Uncertainty on Pavlovian and Instrumental Generalization Slopes**

|  | β | 95% CI (Lower) | 95% CI (Upper) | *p* | η_p_^2^ |
| --- | --- | --- | --- | --- | --- |
| RR | 0.01 | -0.02 | 0.03 | .621 | <.01 |
| R. AI | -0.22 | -0.58 | 0.13 | .218 | <.01 |
| L. AI | -0.18 | -0.56 | 0.17 | .313 | <.01 |
| dmPFC | -0.25 | -0.66 | 0.16 | .231 | <.01 |
| L. vmPFC | -0.10 | -0.54 | 0.34 | .259 | <.01 |
| Avoidance | 0.48 | -0.90 | 2.06 | .442 | <.01 |

Stimulus * Intolerance of uncertainty interaction effects are reported for each task measure, to indicate the effect of intolerance of uncertainty on generalization slopes. Intolerance of uncertainty scores are scaled and mean-centered. RR: risk rating; R.: right; L.: left; AI: anterior insula; dmPFC: dorsomedial prefrontal cortex; vmPFC: ventromedial prefrontal cortex.

**Table S8. Correlations between Indices of Pavlovian and Instrumental Generalization**

|  |  | *r* | *p* |
| --- | --- | --- | --- |
| GS_avg_ Avoid | GS_avg_ RR | 0.42 | <.001 *** |
|  | GS_avg_ R. AI | 0.13 | .133 |
|  | GS_avg_ L. AI | 0.11 | .217 |
|  | GS_avg_ dmPFC | 0.26 | .002 ** |
| CS+ Avoid | CS+ RR | 0.31 | <.001 *** |
|  | CS+ R. AI | 0.23 | .007 ** |
|  | CS+ L. AI | 0.22 | .012 * |
|  | CS+ dmPFC | 0.33 | <.001 *** |

Pearson’s *r* reported; *df*=144 for analyses with behavioral variables only, *df*=133 for analyses with fMRI variables. Corresponding △CS- values were subtracted from all GS_avg_ and CS+ values. RR: risk rating; R.: right; L.: left; AI: anterior insula; dmPFC: dorsomedial prefrontal cortex; vmPFC: ventromedial prefrontal cortex.

**Table S9. Moderating Effect of vmPFC on APIC**

|  | β | 95% CI Lower | 95% CI Upper | *p* | η_p_^2^ |
| --- | --- | --- | --- | --- | --- |
| GS_avg_ Predictors of GS_avg_ Avoidance |  |  |  |  |  |
| RR x L. vmPFC | -2.68 | -13.31 | 7.94 | .618 | <0.01 |
| R. AI x L. vmPFC | -4.27 | -10.3 | 1.76 | .164 | 0.01 |
| L. AI x L. vmPFC | -6.04 | -11.27 | -0.81 | .024 * | 0.04 |
| dmPFC x L. vmPFC | -7.08 | -11.93 | -2.23 | .005 ** | 0.05 |
| CS+ Predictors of CS+ Avoidance |  |  |  |  |  |
| RR x L. vmPFC | 2.67 | -23.76 | 29.1 | .842 | <0.01 |
| R. AI x L. vmPFC | -3.4 | -13.43 | 6.63 | .503 | <0.01 |
| L. AI x L. vmPFC | -4.65 | -13.65 | 4.35 | .308 | 0.01 |
| dmPFC x L. vmPFC | -4.38 | -13.93 | 5.16 | .365 | <.001 |

Models examined interaction of Pavlovian fear indices with vmPFC to predict avoidance, e.g. *GS_avg_ Avoidance ~ GS_avg_ RR x GS_avg_ L. vmPFC*. Corresponding △CS- values were subtracted from all GS_avg_ and CS+ values. RR: risk rating; R.: right; L.: left; AI: anterior insula; dmPFC: dorsomedial prefrontal cortex; vmPFC: ventromedial prefrontal cortex.

**Table S10. Moderating Effect of Anxiety-Related Disorder Status on APIC**

|  | β | 95% CI Lower | 95% CI Upper | *p* | η_p_^2^ |
| --- | --- | --- | --- | --- | --- |
| GS_avg_ Predictors of GS_avg_ Avoidance |  |  |  |  |  |
| RR x Dx | 1.43 | -7.28 | 10.13 | .747 | <0.01 |
| R. AI x Dx | 8.61 | 1.12 | 16.1 | .025 * | 0.04 |
| L. AI x Dx | 6.25 | -1.8 | 14.31 | .127 | 0.02 |
| dmPFC x Dx | 4.96 | -1.9 | 11.82 | .155 | 0.01 |
| CS+ Predictors of CS+ Avoidance |  |  |  |  |  |
| RR x Dx | 12.80 | -16.11 | 41.70 | .383 | <0.01 |
| R. AI x Dx | 5.85 | -9.61 | 21.30 | .456 | <0.01 |
| L. AI x Dx | 1.31 | -13.8 | 16.43 | .864 | <0.01 |
| dmPFC x Dx | 7.42 | -6.24 | 21.08 | .284 | 0.01 |

Models examined interaction of Pavlovian fear indices with diagnostic group to predict avoidance, e.g. *GS_avg_ Avoidance ~ GS_avg_ RR x Dx.* Corresponding △CS- values were subtracted from all GS_avg_ and CS+ values. RR: risk rating; R.: right; L.: left; AI: anterior insula; dmPFC: dorsomedial prefrontal cortex; vmPFC: ventromedial prefrontal cortex; Dx: diagnosis (patient versus healthy comparison).

**Table S11. Moderating Effect of Anxiety Sensitivity on APIC**

|  | β | 95% CI Lower | 95% CI Upper | *p* | η_p_^2^ |
| --- | --- | --- | --- | --- | --- |
| GS_avg_ Predictors of GS_avg_ Avoidance |  |  |  |  |  |
| RR x AS | 4.02 | -6.41 | 14.45 | .447 | <0.01 |
| R. AI x AS | 12.42 | 2.02 | 22.81 | .020 * | 0.04 |
| L. AI x AS | 12.35 | 1.08 | 23.62 | .032 * | 0.03 |
| dmPFC x AS | 7.50 | -1.61 | 16.61 | .106 | 0.02 |
| CS+ Predictors of CS+ Avoidance |  |  |  |  |  |
| RR x AS | 25.05 | -15.01 | 65.1 | .219 | 0.01 |
| R. AI x AS | 9.16 | -9.89 | 28.21 | .343 | 0.01 |
| L. AI x AS | 7.76 | -10.67 | 26.2 | .406 | <0.01 |
| dmPFC x AS | 5.79 | -11.06 | 22.64 | .498 | <0.01 |

Models examined the interaction of Pavlovian fear indices with anxiety sensitivity to predict avoidance, e.g. *GS_avg_ Avoidance ~ GS_avg_ RR x AS.* Corresponding △CS- values were subtracted from all GS_avg_ and CS+ values. RR: risk rating; R.: right; L.: left; AI: anterior insula; dmPFC: dorsomedial prefrontal cortex; vmPFC: ventromedial prefrontal cortex; AS: anxiety sensitivity.

**Table S12. Moderating Effect of Intolerance of Uncertainty on APIC**

|  | β | 95% CI Lower | 95% CI Upper | *p* | η_p_^2^ |
| --- | --- | --- | --- | --- | --- |
| GS_avg_ Predictors of GS_avg_ Avoidance |  |  |  |  |  |
| RR x IU | 4.64 | -16.99 | 26.28 | .672 | <0.01 |
| R. AI x IU | 13.14 | -7.26 | 33.55 | .205 | 0.01 |
| L. AI x IU | 16.90 | -3.25 | 37.06 | .100 | 0.02 |
| dACC. dmPFC x IU | 10.15 | -7.37 | 27.66 | .254 | 0.01 |
| CS+ Predictors of CS+ Avoidance |  |  |  |  |  |
| RR x IU | 20.82 | -45.35 | 86.99 | .535 | <0.01 |
| R. AI x IU | 14.37 | -22.06 | 50.80 | .437 | <0.01 |
| L. AI x IU | 10.72 | -23.31 | 44.74 | .534 | <0.01 |
| dACC. dmPFC x IU | 16.99 | -15.83 | 49.81 | .308 | 0.01 |

Models examined the interaction of Pavlovian fear indices with anxiety sensitivity to predict avoidance, e.g. *GS_avg_ Avoidance ~ GS_avg_ RR x IU.* Corresponding △CS- values were subtracted from all GS_avg_ and CS+ values. RR: risk rating; R.: right; L.: left; AI: anterior insula; dmPFC: dorsomedial prefrontal cortex; vmPFC: ventromedial prefrontal cortex; AS: anxiety sensitivity.

Table S13. Neural Predictors of Choice

| Window of neural activity | Cluster name | Cluster size (voxels) | Peak coordinates | | | Peak *d’* | IU correlation with peak *d’* | AS correlation with peak *d’* | AD vs HC  comparison of peak *d’* | |
| --- | --- | --- | --- | --- | --- | --- | --- | --- | --- | --- |
|  |  |  | X | Y | Z | Mean (SD) | *r* | | *df* | *t* |
| Entire imaginal period | Med. Occip. | 1259 | 52 | 25 | 44 | 1.07 (0.36) | .04 | .01 | 76.99 | 0.52 |
|  | L. Frontal Pole | 189 | 33 | 96 | 45 | 0.84 (0.41) | -.09 | -.15 | 73.46 | -0.11 |
|  | L. SPL | 1091 | 36 | 37 | 72 | 1.06 (0.3) | -.09 | -.08 | 78.68 | 1.38 |
|  | R. SPL | 182 | 74 | 37 | 65 | 0.80 (0.33) | -.13 | -.03 | 63.09 | 1.99 |
|  | L. M1 | 1274 | 24 | 57 | 68 | 1.12 (0.38) | -.16 | -.18 | 82.66 | 0.08 |
|  |  |  |  |  |  |  |  |  |  |  |
| Imagine-avoid | Med. Occip. | 842 | 48 | 26 | 41 | 0.99 (0.39) | .06 | .03 | 84.84 | 0.16 |
|  | L. M1 | 1206 | 25 | 57 | 68 | 1.11 (0.35) | -.25* | -.20 | 77.17 | 1.07 |
|  | L. SPL | 595 | 42 | 33 | 72 | 0.94 (0.29) | -.08 | -.03 | 73.16 | 1.21 |
|  |  |  |  |  |  |  |  |  |  |  |
| Imagine-approach | Med. Occip. | 1252 | 52 | 23 | 44 | 1.07 (0.34) | -.12 | -.06 | 75.22 | 1.32 |
|  | dmPFC | 151 | 50 | 78 | 61 | 0.77 (0.35) | -.06 | -.12 | 76.69 | 0.17 |
|  | Sup. Pcu. | 331 | 52 | 35 | 67 | 0.87 (0.32) | -.16 | -.05 | 71.37 | 0.63 |
|  | R. IPS | 180 | 73 | 42 | 65 | 0.78 (0.32) | -.25* | -.15 | 78.21 | 0.41 |
|  | L. SPL | 518 | 40 | 35 | 73 | 0.92 (0.33) | -.18 | -.05 | 78.27 | 1.34 |
|  | L. M1 | 178 | 24 | 57 | 68 | 0.79 (0.38) | -.17 | -.15 | 84.34 | 0.24 |

Med.: medial; Occip.: occipital; L.: left; R.: right; Sup. Pcu: superior precuneus; SPL: superior parietal lobule; M1: primary motor cortex; dmPFC: dorsomedial prefrontal cortex; IPS: intraparietal sulcus. Correlations and t-tests reflect peak predictive accuracy within cluster. **p* <.05.

**Figure S1.** Generalization of Pavlovian activity in left ventromedial prefrontal cortex.

ΔCS-: triangular conditioned safety-cue; oCSm: ring-shaped conditioned safety-cue; GS2 and GS3: generalization stimulus classes 2 and 3; CS: conditioned danger-cue. Bolded diamonds reflect significant differences from CS+ based on post hoc *t*-test comparisons.

Figure S2. Effects of diagnostic status on Pavlovian and instrumental generalization

tCSm: triangular conditioned safety-cue; oCSm: ring-shaped conditioned safety-cue; GS2 and GS3: generalization stimulus classes 2 and 3; CSp: conditioned danger-cue. Bolded diamonds reflect significant differences from tCS- (rows 1-5) or CS+ (row 6) based on post hoc *t*-test comparisons.

**Figure S3.** Interaction of generalized left anterior insula activity , generalized left vmPFC activity, and anxiety sensitivity on generalized avoidance.

vmPFC: ventromedial prefrontal cortex; ASI: Anxiety Sensitivity Index; AI: anterior insula; Gen.: generalization. Neural or behavioral responses to the triangular safety-cue were subtracted from all Gen. indices.

**Figure S4.** Neural activity corresponding to mental simulation.

**Figure S5.** Neural activity during mental simulation predicting avoidance decisions.

Med.: medial; Occip.: occipital; L.: left; R.: right; Sup. Pcu: superior precuneus; SPL: superior parietal lobule; M1: primary motor cortex; dmPFC: dorsomedial prefrontal cortex; IPS: intraparietal sulcus. Scatterplots show subject-averaged and group-averaged activity to “imagine-approach” (horizontal axis) and “imagine-avoid” (vertical axis) preceding approach decisions (blue points) and avoidance decisions (red points).
